# Evaluation of YouTube Bariatric Surgery Videos in the Context of Medical Tourism

**DOI:** 10.7759/cureus.44642

**Published:** 2023-09-04

**Authors:** Suleyman Caglar Ertekin

**Affiliations:** 1 General Surgery, Private Practice, Izmir, TUR; 2 General Surgery, Altınbas University, Istanbul, TUR

**Keywords:** discern score, jama score, youtube™, bariatric surgery, medical tourism

## Abstract

Introduction

The increasing prevalence of obesity has led to the popularity of bariatric and metabolic surgery, often sought through medical tourism due to constraints within public healthcare systems. This study aimed to examine the quality and impact of YouTube videos related to bariatric surgery within the context of medical tourism.

Materials and methods

In June 2023, a YouTube search for "Bariatric Surgery Medical Tourism" and "Obesity Surgery Medical Tourism" yielded the top 200 videos, from which 33 were chosen after applying exclusion criteria. These videos underwent further screening based on source, duration, and content. Quality was assessed using established scales, including the Journal of American Medical Association (JAMA) criteria, the Global Quality Scale (GQS), and modified DISCERN score.

Results

Thirty-three videos were chosen for comprehensive analysis. Among the videos, 48.5% portrayed patient experiences in the context of medical tourism bariatric surgery, providing valuable insights. The videos had varying durations and engagement metrics, with an average GQS score of 2.09, JAMA score of 2.57, and DISCERN score of 3.06. Notably, videos depicting patient experiences had distinct characteristics and higher evaluation scores, emphasizing their significance within the study.

Conclusion

This study assessed YouTube videos related to bariatric surgery within the realm of medical tourism. The research illuminated diverse facets of medical tourism concerning obesity surgery and the quality of information disseminated on YouTube. Although patient experience videos received higher quality ratings, the overall reliability and content diversity underscored the potential and challenges of utilizing YouTube as an information source for medical tourism.

## Introduction

Bariatric surgery presently maintains its position as the most potent intervention for morbid obesity. Its increasing popularity corresponds to the escalating prevalence of obesity, a trend underscored by the findings of the World Health Organization (WHO) [1].

However, the prevalence of obesity and morbid obesity creates a significant burden that surpasses the annual count of surgical and endoscopic interventions. The efficacy of interventions faces additional complexities due to limitations within public healthcare systems, causing certain patients to rely on services offered by the private sector within their respective countries, frequently resulting in significant financial burdens. Consequently, certain patients are turning to medical tourism, an outcome of globalization, as a means to address their obesity-related issues [2]. YouTube can also emerge as a research source. Due to the extensive utilization and convenient accessibility of the internet, YouTube has gained recognition as a fundamental reference source for health-related information. Still, the lack of peer-review and the diverse group of content creators on YouTube have led to the spread of unreliable health information. This concerns the reliability of health tourism details [3-5]. Moreover, YouTube videos are now a vital source for medical students and surgeons. Technological advancements have given rise to a novel YouTube generation, influenced by the merits and demerits of information dissemination and the questionable quality of these videos [6, 7].

Patients are increasingly using YouTube for medical insights. Yet, the abundance of incomplete and misleading content poses significant challenges. Studies reveal that medical information, including YouTube videos, often delivers inaccurate and unsuitable material [2, 8-10]. This negatively affects the patient-doctor relationship. Notably, 38% of physicians perceive that patient consultations have become less effective due to information gathered from various sources, potentially influencing patients' decision-making processes [11]. The surge in bariatric surgeries and the increasing interest in bariatric procedures have resulted in a notable uptick in the dissemination of shared bariatric surgical practices and information related to medical tourism. This trend is particularly evident on online platforms. The objective of this study was to assess the quality of educational videos concerning medical tourism-linked bariatric surgery accessible on YouTube, while also examining their potential influence on viewers. This research stands out as one of the initial inquiries within the literature to delve into this specific subject.

## Materials and methods

Study design

In June 2023, a search query for the terms "Bariatric Surgery Medical Tourism" and "Obesity Surgery Medical Tourism" was entered into the search field of the YouTube platform using the English language setting. The search was conducted with the default filters applied. The videos were evaluated by a general surgeon who performs over 100 medical tourism-based obesity surgeries from abroad annually. Subsequently, the first 200 videos ranked by the YouTube algorithm were meticulously evaluated.

Inclusion and exclusion criteria

Within the scope of the research, the defined exclusion criteria are as follows: duplicated content, videos with a duration of less than 1 minute, advertisement-oriented videos, videos lacking relevance that appear in YouTube search results but are completely unrelated to medical tourism and obesity surgery, videos solely focused on personal illness experiences, and videos not in the English language. The inclusion criteria encompassed thematic relevance, general accessibility, and a minimum duration of 1 minute.

Data collection

The subsequent investigation encompassed the documentation and subsequent analysis of diverse video attributes, video origin, intended audience, duration, viewership metrics (view count and likes), and time elapsed since the initial upload. The videos' origins were categorized into distinct groups, including private hospitals or organizations, physicians, health information websites, and independent contributors.

The evaluation of the videos entailed the utilization of three established standard assessment scales commonly applied for content evaluation. To assess video credibility, the JAMA 4-criteria benchmarks were utilized. The quality of educational content in the videos was gauged using the Global Quality Scale (GQS). Additionally, the modified DISCERN score was applied to examine both the reliability and quality dimensions of the videos (Table 1) [12-16].

**Table 1:** Standard scales used for evaluation of YouTube videos. JAMA: The Journal of the American Medical Association; GQS: The Global Quality Scale

The Journal of the American Medical Association (JAMA) establishes evaluation criteria (1 point for each criterion, yielding a total score of 4 points).	The Global Quality Scale (GQS) assigns scores on a range from 1 (indicating poor quality) to 5 (indicating excellent flow and quality).	The Modified DISCERN employs a scoring mechanism where 1 point is attributed for each "Yes" and 0 points for each "No."
Authorship: Information regarding the credentials and affiliations of authors and contributors must be furnished.	The video exhibits low quality, lacks a coherent structure, lacks substantial information, and offers minimal utility to patients. Score: 1	Does the video exhibit clarity, conciseness, and comprehensibility?
Attribution: Presents copyright details comprehensively and cites references and sources for the content.	The video displays an overall subpar quality and lacks a smooth presentation. While some information is included, numerous crucial topics are absent, resulting in limited value for patients. Score: 2	Does the video rely on credible sources of information? (e.g., citing publications, featuring specialist speakers)
Currency: The original posting date of the content and any subsequent updates should be supplied.	The video demonstrates a moderate level of quality, although its flow could be improved. While certain important information is sufficiently addressed, other aspects are inadequately covered, leading to moderate usefulness for patients. Score: 3	Does the information provided maintain a balanced and impartial perspective?
Disclosure: Any potential conflicts of interest, funding sources, sponsorships, advertising affiliations, support, and ownership of the video need to be completely revealed.	The video showcases a commendable quality with a smooth flow. It effectively includes the majority of pertinent information, yet some topics remain unaddressed. The video proves valuable for patients. Score: 4	Are supplementary information sources provided for patient reference?
	The video is characterized by exceptional quality and seamless flow, offering significant utility to patients. Score: 5	Are any areas of uncertainty or controversy acknowledged?

This study involved the analysis of publicly available online videos intended for a general audience, using descriptive research. Since the videos did not involve patient-related data and were openly accessible on YouTube.com, there was no need for institutional review board or ethics committee approval.

Statistical analysis

Baseline clinical data underwent rigorous statistical analysis. For continuous data, t-tests and Mann-Whitney U analyses were conducted, while categorical data were analyzed using Fisher's exact test or the chi-square test. SPSS software version 22.0 (IBM Corp., Armonk, NY, USA) was used for analysis. All tests were two-tailed, and significance was set at P < 0.05.

## Results

**Figure 1:**
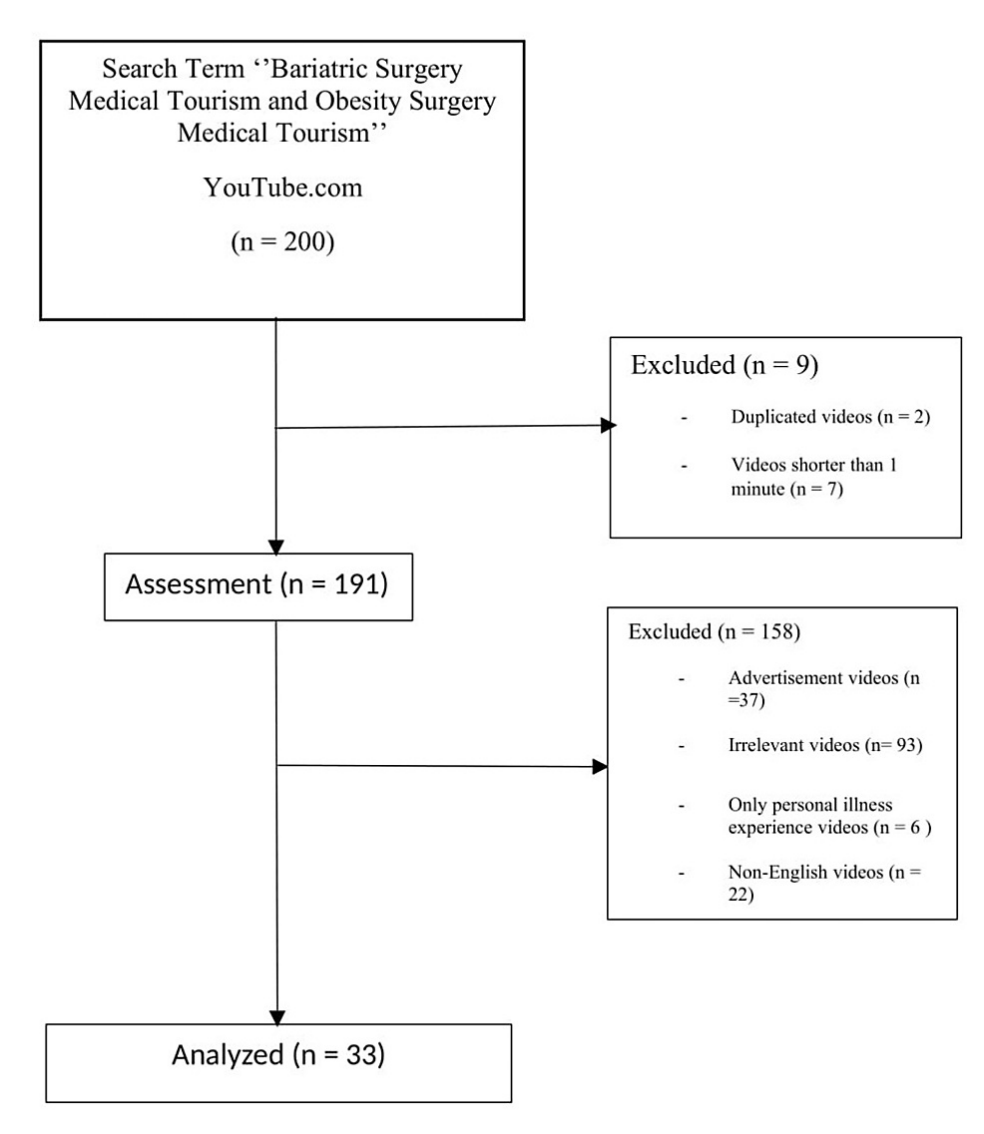
Flowchart of video selection.

Thirty-three videos meeting the study's criteria were selected for in-depth analysis, as depicted in Figure 1. The videos originated from diverse sources: private hospitals/organizations (48.5%), physicians (15.2%), health information websites (15.2%), and independent users (21.2%). Video uploads were primarily from Mexico (54.5%), followed by the United States (15.2%), India (18.2%), Turkey (9.1%), and Costa Rica (3%).

Among the videos, 16 (48.5%) depicted patient experiences, providing insights into medical tourism bariatric surgery. Median video duration was 5.19 minutes (range: 1.10-53.40), with likes ranging from 0 to 183 and a median view count of 1784 (range: 297-33,678). The median time since the video upload was 1869 days (range: 90-5972). Evaluation scores included a mean GQS score of 2.09 ± 0.45, JAMA score of 2.57 ± 0.86, and modified DISCERN score of 3.06 ± 1.22 (Table 2).

**Table 2:** Detailed characteristics of medical tourism bariatric surgery videos on YouTube JAMA: The Journal of the American Medical Association; GQS: The Global Quality Scale

Parameters	N = 33 (%)
Video source	
-Private hospital/organizations	16 (48.5)
-Physicians	5 (15.2)
-Health information websites	5 (15.2)
-Independent Users	7 (21.2)
Country of Video Upload Source	
-United States of America	5 (15.2)
-Mexico	18 (54.5)
-India	6 (18.2)
-Turkey	3 (9.1)
-Costa Rica	1 (3)
Videos Depicting Patient Experiences	16 (48.5%)
Duration (median, min) (min-max)	5.19 (1.10 – 53.40)
Likes (median, min) (min-max)	5 (0 – 183)
Views (median, min) (min-max)	1784 (297 – 33678)
Duration since video upload date, days (median) (min-max)	1869 (90 – 5972)
GQS score (mean-SD) (min-max)	2.09 ± 0.45 (1 – 3)
JAMA score (mean-SD) (min-max)	2.57 ± 0.86 (1 – 4)
DISCERN score (mean-SD) (min-max)	3.06 ± 1.22 (0 – 5)

Video characteristics were analyzed for various sources. Median durations were: private hospitals/organizations (5.25 minutes), physicians (7.65 minutes), health information websites (3.48 minutes), and independent users (15.30 minutes). Likes and view counts varied across sources, but differences were not statistically significant (p > 0.05). Duration since upload also varied (p > 0.05). GQS scores showed no significant differences (p > 0.05), while JAMA and modified DISCERN scores significantly differed across sources (p < 0.001) (Table 3).

**Table 3:** Assessment of video sources based on video characteristics JAMA: The Journal of the American Medical Association; GQS: The Global Quality Scale

Video features	Private hospital/organizations	Physicians	Health information websites	Independent users	P-value
Duration (median) (min-max)	5.25 (1.35 – 53.40)	7.65 (1.12 – 9.52)	3.48 (1.10 – 4.45)	15.30 (1.45 – 51.58)	0.079
Likes (median) (min-max)	5 (0 – 183)	9 (0 – 29)	4 (1 – 170)	9 (0 – 91)	0.810
Views (median) (min-max)	2251 (297 – 33678)	1500 (496 – 2589)	620 (422 – 23789)	2530 (324 – 15906)	0.467
Duration since video upload date, days (median) (min-max)	2473 (90 – 5972)	1460 (450 – 5110)	1825 (324 – 3201)	1095 (367 – 5436)	0.661
GQS score (mean ± SD)	2.12 ± 0.08	2.2 ± 0.20	2 ± 0.31	2 ± 0.21	0.852
JAMA score (mean ± SD)	2.62 ± 0.22	2.8 ± 0.37	2.40 ± 0.50	2.42 ± 0.29	0.864
DISCERN score (mean ± SD)	3.06 ± 0.28	3.6 ± 0.24	2.40 ± 0.92	3.14 ± 0.40	0.501

Videos depicting patient experiences (n = 16) had a median duration of 5.39 minutes, while other videos (n = 17) had a median duration of 3.49 minutes. Significant differences in JAMA and modified DISCERN scores were observed between the two categories (p < 0.001) (Table 4).

**Table 4:** Evaluation of video sources depicting patient experiences JAMA: The Journal of the American Medical Association; GQS: The Global Quality Scale

	Videos Depicting Patient Experiences (n = 16), (48.5%)	Other Videos (n = 17), (51.5%)	P-value
Duration (median) (min-max)	5.39 (1.10 – 23.22)	3.49 (1.12 – 53.40)	0.197
Likes (median) (min-max)	5.5 (0 – 91)	5 (0 – 183)	0.445
Views (median) (min-max)	1607 (297 – 21977)	2100 (324 – 33678)	0.065
Duration since video upload date, days (median) (min-max)	1970 (332 – 5972)	1560 (90 – 5436)	0.743
GQS score (mean±SD)	2.12 ± 0.08	2.05 ± 0.13	0.685
JAMA score (mean±SD)	3.32 ± 0.11	1.88 ± 0.11	< 0.001
DISCERN score (mean±SD)	3.93 ± 0.14	2.23 ± 0.26	< 0.001

## Discussion

This study represents the initial endeavor to assess the content presented in YouTube videos related to bariatric surgery within the context of medical tourism. The investigation delved into answering queries regarding various aspects of obesity surgery medical tourism, encompassing patients' preparatory measures during the preoperative phase, methods of patient reception upon arrival, intricate details of the surgical procedures, postoperative rehabilitation processes, and protocols for patient return and follow-up. In this context, the study focused on the informative content disseminated through videos hosted on the YouTube platform, with a specific emphasis on the sequential stages of services rendered in the domain of obesity surgery under the umbrella of medical tourism.

A notable proportion of the videos originated from Mexico (54.5%). This observation might allude to Mexico's significance as a prominent destination for obesity surgery medical tourism. These findings align with prior research efforts in the same field [17, 18].

In our study, we evaluated the information conveyed in the videos and determined an overall moderate quality of the videos. These findings may indicate the diversity of sources and content in medical tourism videos related to bariatric surgery. While a significant portion of the videos centered around patients' experiences, they also provided general insight into the quality and content of the videos. Our study did not reveal any significant disparities in reliability and quality among private organizations, physicians, health information websites, and independent users. However, these results stand in contrast to several previous studies that have examined patient information videos on YouTube [19, 20].

In our study, we employed the three most common scoring systems, and a positive association was observed among these systems. These findings highlight that videos portraying patients' experiences are linked with higher JAMA and modified DISCERN scores, indicative of superior quality. These results underscore the potential of videos encompassing individual patient experiences to offer more comprehensive and qualitatively enriched information regarding medical tourism surgeries. However, these outcomes diverge from the prevailing consensus in the literature, demonstrating that user-generated videos, alongside those produced by healthcare professionals or institutions, exhibit comparable levels of reliability and quality. Additionally, upon scrutinizing videos categorized as "Video Sources Depicting Patient Experiences" those featuring patients sharing their experiences of medical tourism for obesity surgery emerged as a more extensive informational resource. This distinction may arise from medical tourism encapsulating not only health-related dimensions but also other facets directly experienced by individuals [14, 16, 21].

This study has some limitations. Our assessments might be considered superficial due to the lack of an established tool for evaluating video data on a specific topic. Furthermore, the videos were located utilizing YouTube's default search settings. The outcomes of the search can be influenced by factors such as relevance, engagement, and quality, all of which may differ among users. Therefore, extrapolating the conclusions of this cross-sectional study to encompass all users may present challenges. In addition, our evaluations were carried out by a sole surgeon, and appraisals conducted by diverse healthcare professionals or experts could potentially yield disparate results.

## Conclusions

The study examined various aspects of medical tourism related to bariatric surgery within the context of obesity management, along with the quality of information shared on YouTube. It was observed that patient experience videos garnered higher quality ratings. Nevertheless, limitations pertaining to overall reliability and content diversity underscored the potential and challenges of YouTube as an informational resource for medical tourism.
